# Supplementary material for: Effects of Short-Term Lenvatinib Administration Prior to Transarterial Chemoembolization for Hepatocellular Carcinoma
Source: Cancers (Basel). 2024 Apr 23;16(9):1624. doi: 10.3390/cancers16091624 (PMC11083824; doi:10.3390/cancers16091624)
Supplement: Supplementary file 1 [file cancers-16-01624-s001.zip › cancers-2968500-supplementary.pdf]

Table S1. The clinical characteristics of patients undergoing cTACE or DEB-TACE in short-term LEN-TACE.

**Characteristic**	**cTACE (*n* = 19)**	**DEB-TACE (*n* = 6)**	***P* value**
Age, median (range), years	79 (63-90)	84.5 (68-89)	0.48
Weight, median (range), kg	59.2 (44.5-89.8)	57.3 (45.0-65.1)	0.23
Sex Male, %	13 (68.4)	5 (83.3)	0.64
Etiology, %			
Hepatitis B	1 (5.3)	1 (16.7)	0.18
Hepatitis C	7 (36.8)	0 (0)	
Non-B non-C	11 (57.9)	5 (83.3)	
Child-Pugh stage, %			
A	18 (94.7)	4 (66.7)	0.13
B	1 (5.3)	2 (33.3)	
mALBI grade, %			
1,2a	13 (68.4)	3 (50.0)	0.63
2b	6 (5.3)	3 (50.0)	
AFP , %			
<200 ng/mL	16 (84.2)	4 (66.7)	0.73
≥200 ng/mL	3 (15.8)	2 (33.3)	
tumor size, median (range), mm	21 (11-63)	73.5 (12-108)	<0.05*
tumor number, median(range)	1 (1-4)	1 (1-15)	0.78
Liver cancer staging, %			
early	14 (73.7)	1 (16.7)	<0.05*
intermeidate	5 (26.3)	5 (83.3)	

Table S2. Univariate analyses of clinical characteristics associated with CR rate and PFS.

Variable	CR rate (4 weeks after the first TACE)		PFS	
	OR(95%CI)	*p* value	HR(95%CI)	*p* value
Etiology(virus / non-virus)	0.875(0.116-6.579)	0.897	2.021(0.225-18.146)	0.530
Child-Pugh(A / B)	0.471(0.034-6.568)	0.575	0.408(0.045-3.706)	0.426
mALBI(1,2a / 2b)	0.875(0.116-6.579)	0.897	0.390(0.065-2.350)	0.304
AFP(<200ng/ml / ≧200ng/ml)	1.067(0.092-12.401)	0.959	0.226(0.035-1.452)	0.117
Tumor size(<50mm / ≧50mm)	0.078(0.008-0.792)	0.031	0.438(0.043-4.463)	0.486
Tumor number(1 / ≧2)	0.917(0.123-6.825)	0.932	1.702(0.283-10.240)	0.561
Liver cancer staging(eraly / intermediate)	0.089(0.008-1.002)	0.050	0.680(0.112-4.123)	0.675
TACE(cTACE / DEB-TACE)	3.556(0.405-31.233)	0.253	0.3872(0.623-24.047)	0.146
